# Preparing for advanced practice radiation therapy in South Africa: Conceptual and curricular considerations

**DOI:** 10.4102/hsag.v26i0.1587

**Published:** 2021-08-30

**Authors:** Oupa S. Motshweneng, Sibusiso Mdletshe

**Affiliations:** 1 Department of Health Sciences Education, Faculty of Health Sciences, University of Cape Town, Cape Town, South Africa; 2 Department of Anatomy and Medical Imaging, Faculty of Medical and Health Sciences, University of Auckland, Auckland, New Zealand

**Keywords:** curriculum, radiation therapy, training, advanced practice, role extension

## Abstract

The higher education landscape in South Africa has recently changed with the new National Qualifications Framework, leading to the introduction of four-year degrees in all four radiography disciplines. Additionally, there have been developments in the extension of roles in radiography, while the need for interdisciplinary practice has also been emphasised. The Professional Board for Radiography and Clinical Technology (PBRCT) of the Health Professions Council of South Africa is currently revising the scope of the profession which will now include extended roles. However, the extended role concept in radiation therapy has not received the attention similar to Diagnostic/Medical Imaging. The aim of this paper is, therefore, to provide concept clarification and key considerations for developing a postgraduate curricular framework for training radiation therapists to practise in the envisaged extended roles. For this narrative review, a Boolean search for advanced practice and role extension in radiation therapy was done on all databases (43) available on EbscoHost to source for peer-reviewed articles published between January 1950 and September 2020. A total of 17 articles met the inclusion criteria and were used to frame the discussion. Advanced practice emerged as the more suitable concept as it goes beyond an extension of tasks to include critical characteristics that are necessary to drive transformation in the local social, professional and educational arenas. It is envisaged that some of the key points discussed in this paper could assist the PBRCT in thinking about the implementation of the proposed extended roles for radiation therapists in South Africa.

## Introduction

Radiography education in South Africa (SA) has evolved in the last few years with a focus to have all disciplines of radiography (Diagnostic/Medical Imaging, Nuclear Medicine Technology, Radiation Therapy [RT], and Ultrasound) offered at undergraduate level. Previously, it was mainly medical imaging that was offered at the undergraduate level with the other disciplines available as post-basic qualifications.

Radiation Therapy remains one of the key cancer treatment modalities (Ndlovu 2019) whilst it is estimated that 52% of all cancer patients can benefit from RT (Baskar et al. 2012; Duxbury 2015). However, there is widespread shortage of radiation therapists (RTs) across public cancer treatment centres in SA, and this has a significant bearing on service delivery. This, combined with other factors, has resulted in increased workload for available staff and unacceptable RT waiting times in certain centres (Department of Health 2017). The World Health Organization (WHO) recommends ‘task shifting’ as one of the strategies to tackle staff shortages in health (Harnett et al. 2019). The core of this strategy is to enable healthcare professionals to work beyond their traditional roles (Eddy 2006). Several stakeholders have reported implementing this strategy in response to increasing cancer burden, RT staff shortages and long waiting times (Acharya et al. 2013; Bolderston 2004; Coleman et al. 2014; Eddy 2006; Harnett et al. 2014, 2019; Monk et al. 2013).

Simultaneously, there have been developments in the scope of practice in radiography which have led to altered roles and responsibilities (The College of Radiographers 2003). These changes seem to be because of role extension (RE) and advances in technology. Hardy (undated) defines RE as a post-qualification acquisition of skills, responsibilities and resultant additional professional accountability. Advanced practice (AP) is another concept associated with RE and sometimes RE and AP are used interchangeably (Harnett et al. 2019).

In SA, the Professional Board for Radiography and Clinical Technology (PBRCT) of the Health Professions Council of South Africa (HPCSA) (Department of Health 2020) has drafted a new scope of the profession of radiography, including RT. The recommended scope will allow appropriately trained RTs to undertake roles that were traditionally not performed by them. Whilst processes to approve this scope continue, it is beneficial to discuss how these roles will be developed and implemented. Most radiography-related task-shifting discussions in SA have been in medical imaging, with the concept of RE dominating these discussions (Gqweta 2015; Koch et al. 2017; Munro et al. 2012; Williams 2006; 2009). Internationally, there is paucity of literature on task shifting or RE in RT, a situation not dissimilar to SA.

The aim of this article as a way to contribute to the narrative of RE in RT, especially in the SA context, is twofold: (1) concept clarification, by comparing the concepts of RE and AP, as conceived in RT literature. (2) key considerations for developing a postgraduate curricular framework for training RTs to practise in the envisaged roles.

## Methods

Although the authors borrowed some of the processes of a systematic review (SR), this article is fundamentally a narrative review (NR). Systematic reviews are different from NRs in that they are based on evidence from systematic and comprehensive literature searches in all available resources and minimise selection bias (Pae 2015). Nonetheless, the quality of NRs can be improved by using effective search strategies and some of the processes of a SR aimed at reducing selection bias (Ferrari 2015), as was the case in this project.

A Boolean search was done on all databases (43) available on EbscoHost. The terms ‘advanced practice radiation therapy (APRT)’ OR ‘AP radiotherapy’ OR ‘RT RE’ OR ‘radiotherapy RE’ were used to source for peer-reviewed full-text articles available online and published between January 1950 and September 2020. The expanders were set to ‘apply related words’, ‘apply equivalent subjects’ and ‘also search within the full text of the articles’. The search resulted in 37 articles that were included for potential review.

A second search for the same terms was done on Google Scholar. The search was set to find articles ‘with all of the words’ in the title, published between 1950 and 2020, including patents and citations. The search yielded 51 articles included for potential review, resulting in a total of 88 articles for potential review. Title and abstract scanning and full-text skimming were done using inclusion criteria, and duplicates were removed. After this, a total of 9 articles were included for review. The inclusion criteria were that articles must be full-text, peer-reviewed, available online, published in English, and should discuss some form of ‘task shifting’ in RT.

To actively find work done locally, individual journal searches were done on the South African Radiographer, African Journal of Health Professions Education and Health SA Gesondheid, using the terms ‘AP’ OR ‘RE’ OR ‘radiotherapy’ OR ‘RT’, separately. The searches yielded 132 articles but only 1 was included for review after applying the inclusion criteria and removing duplicates, resulting in a total of 10 articles for review.

A manual search was done on the reference lists of the 10 articles, this process resulted in 5 additional articles, and was repeated until no new articles were found on reference lists of all articles. Lastly, 2 handpicked articles were added to the pool and a final total of 17 articles were included in the review.

### Ethical considerations

This article followed all ethical standards for research without direct contact with human or animal subjects.

## Results

Articles reviewed were published from 2004 to 2020. Advanced practice appears to be the most popular term with 12 articles (70.6%) discussing AP, either alone or with other concepts, whilst RE was discussed in only 2 articles (16.7%). The search focused on AP and RE, but concepts of role development (RD) (2 articles) and role expansion (REx) (2 articles) were noted as they emerged in the literature reviewed. Although the United Kingdom (UK) is generally known to have taken a lead in AP and RE work (Monk et al. 2013), most articles (7) were from Australia, followed by Canada (4). Despite additional efforts to find literature published locally, only 1 article was from Africa and none from SA. A summary of these results is presented in Table 1.

**TABLE 1:** Summary of the results of the narrative review.

Year	Author	Country	Type or methodology	Concepts	Sample or focus	Findings or conclusion
2004	Bolderston	Canada	Qualitative: Literature review	AP	International and local trends	AP RTs have been successful in several countries and are currently evolving in Ontario, Canada.
2005	White et al.	Hong Kong	Qualitative: Survey and interviews	Role development,expansion or extension	Clinical oncology professionals at four oncology departments in local public hospitals.	Role development in RT should relieve departmental workloads. Inter-professional collaboration is necessary to ensure maximum benefits from role development.
2006	Eddy	United Kingdom	Qualitative: A discussion paper drawing from a literature review	AP or RE, role development	Literature on advanced practice, expert practice and expert practice in RT, locally and internationally.	AP can and should include elements of RE and development. There is a need for collaboration between stakeholders to develop curricula that will support existing and emerging APRT roles.
2007	Martino and Odle	United States of America	Special report on activities, research and findings of an expert panel.	AP or RE, role expansion	International and local developments	Well planned establishment of AP RTs’ roles can lead to improved efficiencies, patient satisfaction and care and new professional developments for RTs. Open dialogue between stakeholders is critical.
2009	Acharya et al.	Australia	Qualitative: Systematic review	Role expansion	National and international literature on the topic, published between 1990 and 2008.	RT role expansion has been implemented internationally but few reports in Australia. Patient treatment review and image review were the most prominent roles. Despite barriers, positive outcomes are reported.
2011	Kyei and Engel-Hills	Ghana	Qualitative: a case study approach using interviews, participant observation and filed notes.	RE	RTs, a RO and a nurse working at an oncology care centre in Ghana.	The RE resulted in improved quality of care, increased patient satisfaction and reduced RO workloads.
2013	Acharya et al.	Australia	Quantitative: Statistical analysis	Role expansion	8 Radiation Oncologists (ROs), 4 RO registrars 17 RTs	Experienced RTs could assess radiation induced breast skin toxicity as part of their role.
2013	Monk et al.	Australia	Mixed method feasibility study	AP	200 treatment reviews audits, 90 clinical staff surveys at Calvary Mater Newcastle, New South Wales.	Feasibility measures did not reach acceptable levels to support the implementation of advanced practice treatments review roles for RTs at the centre.
2014	Coleman et al.	New Zealand	Mixed methods: A survey	AP	All RTs registered in New Zealand (*n* = 260).	Although there were concerns raised, the implementation of APRT roles was supported by most RTs and the proposal of a master’s degree to train such practitioners was also supported.
2014	Matthews, Wright and Osborne	Australia	A review article describing design, development and implementation of three APRT curricula.	AP	The curriculum was designed by Monash University and piloted at six local cancer clinical sites.	Clinical training integration was successful at the six pilot sites and was subsequently up scaled to 24 sites in the country. Advanced practice RTs’ roles are currently evolving. Lack of standardised training and accreditation remains a challenge.
2014	Harnett et al.	Canada	Article discusses the implementation of APRT role in Ontario. The 8-year project used a mixed methods approach.	AP	Data were sourced from the literature and key stakeholders across Ontario’s cancer centres, using interviews.	APRTs can provide relief to the existing system pressures and allow flexibility in the inter-professional team.
2015	Smith et al.	Australia	A review article	AP	Authors turn to experiences in other countries and professions to describe a conceptual model of AP in RT and diagnostic radiography.	There is a need to develop AP roles that are responding to the needs of the patients and communities. One size does not fit all, contextual factors need to be studied and considered in the development of such roles.
2017	D’Alimonte et al.	Canada	Commentary on the experience of implementing advanced RTs’ roles in a cancer centre	Advanced RTs’ role	The Odette Cancer Centre, Toronto	Addition of advanced RTs’ roles resulted in improved patient waiting times, improved access to services and resulted in an innovative model to deal with increasing complexities.
2018	Hilder, VanDam and Doherty	Australia	A scoping review	AP	Literature discussing APRT roles in Australia	Literature provides limited evidence for the success of APRT roles in Australia. There is more evidence in conference presentations than peer-reviewed articles. More evidence on a national scale is necessary to harness the potential benefits of APRT roles.
2019	Job, Holt and Bernard	Australia	Quantitative	AP	156 palliative patients treated at Radiation Oncology Princess Alexandra Raymond Terrace, Australia	The study found the inter-observer variability between the RO and APRT in defining palliative RT fields to be similar to that reported in the literature between clinicians. Authors justify the establishment of the APRT role.
2019	Harnett et al.	Canada	Qualitative: A feasibility study using literature review and expert consensus	AP	7 APRTs were deployed at four cancer sites to gather information.	There is support of role implementation. Implementation of APRT roles can lead to programme efficiencies and new services, and improved RTs recruitment rates and work satisfaction.
2020	Lim et al.	Singapore	Mixed method: A semi-structured questionnaire	AP	26 ROs and 62 RTs working at the National Cancer Centre in Singapore.	ROs supported the development of the APRT role, whilst RTs called for fairness in role development. Potential benefits include RTs career development, retention and satisfaction and patient satisfaction.

APRT, advanced practice radiation therapy; RT, radiation therapy; RE, role extension; RO, radiation oncologist; AP, advanced practice.

## Discussion

The authors aim to present a discussion by exploring the concepts of RE and AP in RT, and related curricular issues, with an emphasis on the SA context.

### Concept clarification and role definition: Is there still a need?

In SA, the task-shifting discourse is unfolding in the form of RE discussions. Although the need for RE for RTs in SA has been recognised (Lawrence et al. 2011), most discussions are taking place in the medical imaging space (Gqweta 2015; Koch et al. 2017; Munro et al. 2012; Williams 2006; 2009). Moreover, there seems to be no deliberate intentions to define or clarify the concepts of AP and RE in these discussions. A possible reason for this is that unlike internationally where multiple terms are used and potentially cause confusion (Smith et al. 2015), the term RE dominates the task-shifting discourse in SA radiography.

Despite earlier attempts to offer some clarity on the concepts of RE and AP (Hardy & Snaith 2006), authors still report difficulties with role definition (D’Alimonte et al. 2017; Smith et al. 2015), unclarity (Matthews et al. 2014) and confusion (Harnett et al. 2014). It is, therefore, important to attempt to clarify and contextualise these two concepts, that is, RE and AP.

#### Role extension

Eddy (2006) defines RE in RT as the inclusion of a skill or practice area which was previously not part of a typical RTs role. Notably, the included skill or practice area may have been, traditionally, within the role of another profession (Eddy 2006; Martino & Odle 2007; White et al. 2005). This is congruent with the conception of RE by Kyei and Engel-Hills (2011), who studied the possibility of extending the role of RTs in Ghana to include pain assessment, a function typically performed by radiation oncologists (ROs). This also appears to be the way RE is conceived in medical imaging in SA (Gqweta 2015; Koch et al. 2017; Munro et al. 2012; Williams 2006; 2009), and can possibly be interpreted to be the same for RTs in SA, because of the proximity of the disciplines and especially considering that the four new RT extended roles in the PBRCT proposed scope of the profession are traditionally within the scope of ROs and to some degree, the scope of Oncology Nurses (ONs). These roles are: (1) control and administration of contrast media, (2) approval of portal verification images, (3) counselling of patients and their families and (4) on treatment patients review.

#### Advanced practice

It is argued that RE as currently conceived happens in a ‘very practical and skills-based way’ (Harnett et al. 2019), and it is mostly based on delegated tasks (from one profession to the other), which are executed following well-defined protocols (Eddy 2006). Conversely, AP is characterised by functioning at a more autonomous, independent and complex level (Acharya et al. 2009; Bolderston 2004; Harnett et al. 2019; Martino & Odle 2007), with advanced education and training (Martino & Odle 2007; Monk et al. 2013). This is in addition to working beyond traditional boundaries, which may include tasks traditionally performed by ROs (D’Alimonte et al. 2017; Harnett et al. 2014). Such practitioners are expected to be leaders, transformers and innovators (Harnett et al. 2019), both within and outside their particular roles (Coleman et al. 2014).

It is noted that other authors use AP synonymously with REx (Eddy 2006; Harnett et al. 2014), whilst others suggest that AP can have either REx or RE (Martino & Odle 2007) and others argue that it is a synthesis of both (Smith et al. 2015). This confusion appears to lie in the different definitions of REx. Demystifying this is not within the scope of this article. Moreover, REx is a seldom used term in SA radiography, compared to the more popular RE term. Nevertheless, what is clear about AP is that it incorporates the elements of RT RE as it is conceived internationally, in Africa, and most likely in SA, with high level and complex skills and characteristics. The authors recommend further debate with the aim of refining, contextualising and adopting the concept of AP, to develop APRTs who will not only alleviate ROs’ workload by taking additional tasks but will catalyse transformation within and outside their profession through innovation, and new ways of thinking and doing (Harnett et al. 2019).

### Curricular considerations

Education and training significantly impact AP roles as AP revolves around roles that involve new skills (D’Alimonte et al. 2017; Hilder et al. 2013). In the SA context, the PBRCT has set the tone by proposing that extended roles must be preceded by an approved postgraduate qualification whilst noting that there is no prescription of the National Qualifications Framework (NQF) level at which such a qualification must be pitched. Below, the authors discuss some of the fundamental considerations in designing such curricula for SA.

#### The foundation: Philosophies and values

As discussed, and like RE, APRT responds to professional, political and societal needs (Bolderston 2004), but goes further to incorporate critical and high-level characteristics such as leadership, innovation and transformation. Consequently, APRT seems to be better positioned to respond to the need for and aid efforts towards transformation at the multiple local arenas than RE.

If the goal is to train APRTs to be transformers, a transformed and transformative curriculum is needed (Duncan et al. 2006; Frenk et al. 2010) to train transformed transformers, training them to ‘be’ and not just to ‘do’, moving beyond competency alone to form an integrated identity (Jarvis-Selinger et al. 2012). A transformed and transformative curriculum is built on the foundation of rigorously debated values, philosophies and principles (Duncan et al. 2006; Frenk et al. 2010).

#### Programme level

Internationally, all AP articles reviewed (12) agree that some formal education is necessary to prepare RTs for AP. In SA, the PBRCT recommends that this training should be at a postgraduate level. However, the question remains, what type of qualification would this be? An international trend is that a master’s degree is appropriate for preparing RTs for AP (Bolderston 2004; Coleman et al. 2014; Eddy 2006; Martino & Odle 2007; Matthews et al. 2014; Smith et al. 2015). Matthews et al. (2014) particularly recommend a coursework master’s degree. In SA, a master’s degree programme at NQF level 9 appears to be an appropriate vertical articulation as the current minimum undergraduate RT training’s exit level is NQF 8. However, the PBRCT is considering having training for the extended roles offered below a master’s degree level to avoid exclusion of professionals who may only be in possession of the old NQF level 6/7 qualifications.

Horizontal articulation by means of a postgraduate diploma (NQF 8) might seem to be another option. However, according to the national Department of Higher Education and Training (DHET), the postgraduate diploma’s primary purpose is to prepare graduates for highly skilled work by enabling them to ‘undertake advanced reflection and development by means of a systematic survey of current thinking, practice and research methods in an area of specialisation’ (DHET 2013). Based on this description, the postgraduate diploma might be suitable for preparing graduates for RE but not for AP because it does not aim to develop key characteristics of the innovative and transformative leader, which form the foundation of APRT as conceived internationally and contextualised nationally. A master’s degree seems to be a better option as it aims to develop self-directed, original and autonomous graduates who can deal with complex issues in a systematic and creative way (DHET 2013). Nonetheless, stakeholders need to think of ways to ensure that graduates holding the old qualifications have access to the programmes that will be developed. A possible consideration could be to pitch such a programme at level 9 and accommodate those with old NQF levels 6/7 through the recognition of prior learning route or a bridging course.

#### Environment

The environment within which the curriculum unfolds is inextricably linked to the curriculum and is as complex as the curriculum is. This environment includes institutional culture and traditions, people, values, curricular elements and how all these interplay, amongst other things (Genn 2001). Some of the characteristics of a desirable environment include non-hierarchal and non-patriarchal structures and relationships, non-discriminatory policies and processes, interprofessionalism, social justice, gender equity, empowerment, student-centredness, patient-centredness, ethics and enhancement of well-being (Barnes 2007; Genn 2001; Lehmann et al. 2018; McKimm 2007). These must be both taught and lived (Lehmann et al. 2018). It is, therefore, important that stakeholders consider the environment when planning the curriculum, to ensure constructive alignment and minimise the hidden curriculum (unwritten, unofficial and often unintended lessons) (Marsh 2009).

#### Stakeholder engagement

Matthews et al.’s (2014) successful pilot of the APRT curriculum employed stakeholder groups throughout development and implementation. The need for an open dialogue between stakeholders when developing APRT roles and curriculum has also been highlighted by others (Coleman et al. 2014; D’Alimonte et al. 2017; Eddy 2006; Hilder et al. 2013). This is especially important considering that the proposed roles currently fall within the scope of other professions. However, it must also be noted that stakeholders are more than just members of the multidisciplinary team, and other stakeholders, including patients (Harnett et al. 2014), should be consulted using appropriate methods, for the relevant stages of curriculum development. This approach resonates with the primary healthcare (PHC) principles of participation of all, multidisciplinarity and involvement of all service providers (Alperstein 2009). It is also aligned with Beane’s (2001) (as cited by Marsh 2009) conception of a curriculum, which involves many groups and decision-making at various levels, amongst other things. However, a fundamental question remains, who are the stakeholders in the SA context?

In considering the stakeholders, the challenges experienced in developing AP and RE in other countries should be considered, and these include medical dominance (Bolderston 2004; White et al. 2005), professional boundaries (D’Alimonte et al. 2017; Harnett et al. 2014), territorialism (Harnett et al. 2014), ethico-legal issues (Acharya et al. 2009; Eddy 2006; Monk et al. 2013), resources and remuneration (Coleman et al. 2014; Kyei & Engel-Hills 2011), lack of training capacity (Hilder et al. 2013) and management and service restructuring (Hilder et al. 2013; Kyei & Engel-Hills 2011). Considering these challenges, it would seem justifiable to have the multidisciplinary team (RTs, ROs, medical physicists and ONs), health authorities (Department of Health) and regulators (PBRCT), other policymakers (DHET, Department of Employment and Labour), hospital and academic managers, professional bodies and patients as part of the essential stakeholders. This kind of multidisciplinary and multisectoral engagement might assist in navigating some of these challenges.

#### Teaching, learning and assessment

Traditionally, RTs’ training has a strong focus on skills development, aligning with the skills-based nature of the profession. However, there is currently a paradigm shift to training that develops life-long learners. In the SA context, this has been seen with the move to the four-year degree which allows the students to do courses that are beyond the development of clinical skills and now includes courses like research (methodology and project), health education, imaging informatics and management as part of their undergraduate training. This move harmonises well with the principles of APRT.

Advanced practice radiation therapy aims to develop highly skilled professionals who function with more autonomy (Bolderston 2004; Eddy 2006; Harnett et al. 2019; Martino & Odle 2007; Matthews et al. 2014), responsibility and accountability (Acharya et al. 2009; Eddy 2006), more decision-making power and independence (Martino & Odle 2007), who will collaborate with the inter-professional team (D’Alimonte et al. 2017) with professional confidence (Bolderston 2004). It will take more than just a fine selection of content to develop such attributes and characteristics, it will require appropriate pedagogies. It is, therefore, important that when designing the APRT curricula pedagogies are carefully selected, influenced by appropriate learning theories (McKimm 2007). One such learning theory is social constructivism which considers students to be co-constructors of knowledge, where both teachers and students constantly switch positions to facilitate learning (Roth & Radford, as cited in Barker et al. 2015). This type of relationship appears to be conducive to boosting the confidence and collaboration skills of RT students. DasGupta et al. (2006) further argue that non-hierarchical teacher-learner relationships can be translated to professional-patient relationships.

In line with trends in health professions education, more student-centred and active learning methods should be considered for these curricula (McKimm 2007). Such methods’ emphasis on students taking responsibility for their own learning can assist with training more responsible professionals (Harden et al. 1984), a necessary characteristic for APRT. Furthermore, such methods must consider assessment as an important element of the curriculum that is sometimes argued to drive learning (Hift 2014; O’Sullivan et al. 2012). Curricula planners need to carefully choose appropriate methods for assessing the intended competencies for APRT (Epstein 2007; Hift 2014; Schuwirth & Van der Vleuten 2018).

Although the selection of assessment tasks will depend on a clearer and agreed upon role definition and associated competencies, the use of a high stake and all-summative assessment programme should be avoided. However, considering that the qualification will lead to some form of licensure and therefore high stakes assessment tasks cannot be totally avoided, the authors propose a balance of high, medium and low stakes assessments, with both summative and formative tasks and high-quality feedback. This can assist with balancing between educational impact and community or employer confidence.

#### Resources

Resources are an important curricular element; they shape how curricula are planned and enacted (Matthews et al. 2014). Coleman et al. (2014) further report that resources were one of the barriers to implementing APRT roles. Although there are many, one resource factor to consider is expertise, in the form of staff. The question of who will train APRTs, a seemingly controversial debate (Eddy 2006), is particularly significant because some of the tasks that will form part of the envisaged roles belong to other professions.

Based on (1) international trends, (2) SA’s existing undergraduate RTs, ROs and ONs specialist training programmes and (3) alignment with the identified philosophical underpinnings, the authors propose a work-integrated learning (WIL) programme, with an inter-professional approach, facilitated by ROs, ONs and expert RTs (for some AP areas, expert RTs may not be initially available). These three factors are briefly discussed below.

An international trend is the use of a WIL approach (Matthews et al. 2014). In Canada (D’Alimonte et al. 2017) and Australia (Acharya et al. 2009; Matthews et al. 2014), ROs mentored and successfully provided supervision, advanced technical and clinical training to trainees. Matthews et al. (2014) describe how the university taught the theory, whilst ROs mentored and assessed at clinical training sites. This shared curriculum was further supported by clinical facilitators who liaised with the different stakeholders, and an online system where all participants could engage. Locally, WIL is not new as current RT undergraduate programmes employ this approach, and the use of clinical facilitators is common.

Potential gains are that this offering would be in line with the global shift towards inter-professional education (IPE) (McKimm 2007) and can assist in developing inter-professional collaboration and communication, an important characteristic of an APRT and a PHC philosophy principle. Moreover, IPE has been a catalyst for change in modern healthcare (Academy of Science of South Africa 2018). However, this offering will have to be explored with local practicalities in mind. Such practicalities include the fact that in SA, most RT and RO academic training takes place at different types of institutions, for example, out of four institutions that offer RTs training, only one of them also offers ROs training. Moreover, there is a shortage of ROs, who are already responsible for training RO registrars and might not be able to accommodate additional training responsibilities.

The shift to the use of IPE could also consider training collaboration with the nursing profession that already has the AP in the form of ONs. Oncology Nurses curricula include training outcomes that could benefit training of RTs for advanced roles. For example, ON curricula offered within SA include learning outcomes associated with managerial strategies and technologies in an oncology healthcare environment, principles of research in oncology, counselling, etc. Currently, there are at least four universities in SA that offer ON training (Nursing 24 2020). Oncology Nurses clinical facilitators and educators can, therefore, play a crucial role in IPE for shared competencies, especially in allying for the competencies aligned to the extended roles that the PBRCT is considering for implementation. Another consideration to overcome the physical space and resources challenge would be the use of blended learning, which will also increase access by accommodating working professionals who reside far from academic institutions. A number of blended learning programmes have been successfully implemented in SA.

The clinical training component can be led by the respective clinical sites, with ROs providing supervision, RT and ON clinical facilitators providing necessary support. This model has seen success in six Australian centres (Matthews et al. 2014). Moreover, some elements of clinical training can be incorporated into the existing clinical training of RO registrars, to avoid ROs doubling up on work, also considering that tasks (skills) that form part of the proposed extended scope of RTs fall within the remit of ROs and form part of RO registrar training. According to Abratt (2018), there is a relative shortage of ROs in SA and 79.5% (147) of all the country’s ROs are in the private sector. For the proposed model of involving the ROs in RTs training, it would be beneficial to expand the training to include the ROs who are in the private sector. Moreover, there seems to be willingness for such a discussion amongst ROs in SA (Abratt 2018).

#### Programme monitoring and evaluation

Lastly, both the curricula (Marsh 2009) and APRT (Eddy 2006; Matthews et al. 2014) are constantly changing and developing. Therefore, a postgraduate curriculum that is designed to train RTs in AP will need robust monitoring and evaluation mechanisms, to ensure that it remains effective and relevant. Such a review and revision should be based on stakeholder engagements. However, the list of stakeholders needed at this level is not as extended as the one for the initial role development and curriculum design. At this level, essential stakeholders can include students, teachers, academic managers, authorities, service managers and health regulators (McKimm 2007), aligned to the Council on Higher Education (CHE) processes.

**FIGURE 1:**
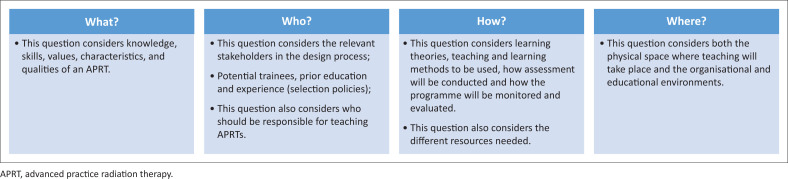
Four broad questions that are essential to facilitate curricula design.

Based on the above discussion and recommendations, four broad questions (Figure 1) are put forward to facilitate the debate on the design and development of the needed curricula.

## Conclusion

This article has explored the concepts of RE and AP as they relate to RT. Advanced practice appears to be the more suitable concept as it goes beyond an extension of tasks, to include critical characteristics that are necessary to drive transformation in the local social, professional and educational arenas. Some fundamental questions that should be considered in designing postgraduate curricula to train SA RTs in AP were also explored. The key points discussed in this article could be instrumental in assisting the PBRCT in strategising and implementing the envisaged RE or AP for RTs in SA.

### Limitations

Although the applied NR approach intentionally aimed to avoid bias that can be associated with NR, this review was not a SR.
